# Prefrontal Physiomarkers of Anxiety and Depression in Parkinson’s Disease

**DOI:** 10.3389/fnins.2021.748165

**Published:** 2021-10-21

**Authors:** Coralie de Hemptinne, Witney Chen, Caroline A. Racine, Andreea L. Seritan, Andrew M. Miller, Maria S. Yaroshinsky, Sarah S. Wang, Roee Gilron, Simon Little, Ian Bledsoe, Marta San Luciano, Maya Katz, Edward F. Chang, Heather E. Dawes, Jill L. Ostrem, Philip A. Starr

**Affiliations:** ^1^Department of Neurological Surgery, University of California, San Francisco, San Francisco, CA, United States; ^2^Department of Psychiatry, University of California, San Francisco, San Francisco, CA, United States; ^3^Department of Neurology, University of California, San Francisco, San Francisco, CA, United States

**Keywords:** Parkinson’s disease, electrocorticography, mood, electrophysiology, prefrontal cortex

## Abstract

**Objective:** Anxiety and depression are prominent non-motor symptoms of Parkinson’s disease (PD), but their pathophysiology remains unclear. We sought to understand their neurophysiological correlates from chronic invasive recordings of the prefrontal cortex (PFC).

**Methods:** We studied four patients undergoing deep brain stimulation (DBS) for their motor signs, who had comorbid mild to moderate anxiety and/or depressive symptoms. In addition to their basal ganglia leads, we placed a permanent prefrontal subdural 4-contact lead. These electrodes were attached to an investigational pulse generator with the capability to sense and store field potential signals, as well as deliver therapeutic neurostimulation. At regular intervals over 3–5 months, participants paired brief invasive neural recordings with self-ratings of symptoms related to depression and anxiety.

**Results:** Mean age was 61 ± 7 years, mean disease duration was 11 ± 8 years and a mean Unified Parkinson’s Disease Rating Scale, with part III (UPDRS-III) off medication score of 37 ± 13. Mean Beck Depression Inventory (BDI) score was 14 ± 5 and Beck Anxiety Index was 16.5 ± 5. Prefrontal cortex spectral power in the beta band correlated with patient self-ratings of symptoms of depression and anxiety, with *r*-values between 0.31 and 0.48. Mood scores showed negative correlation with beta spectral power in lateral locations, and positive correlation with beta spectral power in a mesial recording location, consistent with the dichotomous organization of reward networks in PFC.

**Interpretation:** These findings suggest a physiological basis for anxiety and depression in PD, which may be useful in the development of neurostimulation paradigms for these non-motor disease features.

## Introduction

Anxiety and depression are prominent non-motor symptoms of Parkinson’s disease (PD) that are clinically debilitating (Seppi et al., 2019) and may predate the onset of motor signs (Postuma et al., 2015). Functional imaging studies in patients with PD and comorbid anxiety (Dan et al., 2017; Wang et al., 2017) and depression (Luo et al., 2014; Dan et al., 2017; Wang et al., 2018, 2020; Lin et al., 2020) point to involvement of prefrontal cortical areas, but the associated circuit mechanisms are poorly understood. Much progress has been made in understanding circuit mechanisms related to *motor* signs of PD utilizing invasive intracranial recording at both cortical (Panov et al., 2017) and subcortical (Brittain and Brown, 2014) sites in the motor network. This technique offers much higher spatiotemporal resolution and a more favorable signal-to-noise ratio than most non-invasive methods, and has led to the identification of potential electrophysiological markers for both the severity of motor signs and for the effectiveness of therapeutic intervention (Brittain and Brown, 2014).

Invasive studies in non-parkinsonian disorders have begun to elucidate networks and frequency bands important to mood fluctuations (Kirkby et al., 2018; Sani et al., 2018) and clinical depression (Lipsman et al., 2014; Clark et al., 2016; Merkl et al., 2016; Veerakumar et al., 2019; Scangos et al., 2020). Most invasive human physiological studies have been done perioperatively, using externalized brain leads either during a surgical intervention or for a few days after implantation, in a hospital setting. This method precludes the study of dynamically evolving longitudinal symptoms. Neurostimulation devices that incorporate brain sensing with therapeutic neurostimulation, also called “bidirectional interfaces,” offer many advantages over short term perioperative recordings (Starr, 2018). Advantages include wireless data streaming in real time or from internal device storage, the opportunity to record in fully naturalistic environments, and the possibility of repeated measures of neural activity paired with external monitors, or with patient self-rating of symptoms, over many cycles of symptom exacerbation and remission. A limitation of invasive recording is the sparse spatial coverage, and the inherent risks of inserting a second lead in the context of a clinically indicated surgery. However, despite the minimal risk and wide spatial sampling of non-invasive methods such as scalp electroencephalography and functional magnetic resonance imaging, these non-invasive methods are not suited to performing repeated measures in a patient’s home environment.

To understand prefrontal physiological correlates of anxiety and depression in PD, we studied four patients with PD who met standard clinical criteria for basal ganglia deep brain stimulation (DBS) for their motor signs (Fang and Tolleson, 2017), and who also had comorbid anxiety and/or depressive symptoms. In addition to their standard therapeutic basal ganglia leads, we implanted a permanent quadripolar subdural lead over areas of the right prefrontal cortex (PFC) previously implicated in the pathophysiology of anxiety (Dan et al., 2017; Wang et al., 2017) or depression (Luo et al., 2014; Dan et al., 2017; Wang et al., 2018, 2020; Lin et al., 2020) by non-invasive studies in PD. Daily brain recordings were paired with self-ratings of anxiety and depression. We show that prefrontal oscillatory activity in the beta band, an already well described biomarker of parkinsonian akinesia and rigidity from recordings in the motor system (Brittain and Brown, 2014), predicts anxiety and depressive symptoms in PD. We interpret our results in the framework of a contemporary model of mood regulation, in which an imbalance in reciprocal mesial and lateral prefrontal networks can lead to depression (Rolls, 2016; Loonen and Ivanova, 2017).

## Materials and Methods

This protocol was approved by the Institutional Review Board of the University of California, San Francisco (UCSF), under a physician-sponsored investigational device exemption. Informed consent was obtained under the Declaration of the Principles of Helsinki. The study was registered on ClinicalTrials.gov (NCT03131817)^1^.

### Subjects

Subjects were recruited from the Movement Disorders and Neuromodulation Center at UCSF. Participants had a diagnosis of idiopathic PD and had been offered implantation of a deep brain stimulator system for relief of motor signs. Participants underwent preoperative evaluation by a movement disorders neurologist, a psychiatrist and a neuropsychologist. Motor impairment was assessed using the Unified Parkinson’s Disease Rating Scale, with part III (UPDRS-III) done in the off- and on-medication states (Table 1). Neuropsychological and psychiatric evaluations were conducted using the Montreal Cognitive Assessment, the Beck Anxiety Inventory (BAI), the Beck Depression Inventory (BDI) and the Structured Clinical Interview for DSM-5. Inclusion criteria required mild to moderate depression (BDI > 13) and/or anxiety symptoms (BAI > 7). Patients were excluded for active suicidal ideation on the Columbia Suicidality Severity Rating Scale or significant cognitive impairment (Montreal Cognitive Assessment score <20).

**TABLE 1:** Patient demographics and details of recording.

	Study subject	PD1	PD3	PD4	PD5	Mean and STD
Demographics	Gender	F	M	F	F	
	Age (years)	53	55	67	70	61 ± 7
	Disease duration (years)	25	5	6	9	11.25 ± 8
	Preoperative UPDRS-III (off/on)	39/19	16/15	41/15	50/25	Off: 36.5 ± 14.5 On: 18.5 ± 4.7
	Preoperative depression and anxiety severity (BDI/BAI)	10/16	17/16	9/10	21/24	BDI: 14 ± 5 BAI: 16.5 ± 5
	Preoperative cognitive state (MoCA)	26	29	27	29	27.75 ± 1.5
Details of recording	DBS lead side/target	R and L/STN	R/GP	R/STN	R/GP	
	Cortical region side/target	R/FPC	R/DLPFC	R/OFC	R/OFC	
	Coordinates of recording contact* (contact #: x,y,z)	C8: 18.6 74.6 6.0 C9: 26.9 70.6 1.6	C10: 26.6 80.3 2.4 C11: 33.9 76.6 6.7	C8: 13.4 45.4 15.7 C10: 31.1 13.8 6.9	C8: 18.2 42.6 11.2 C9: 28.3 42.6 9.7	
	#Recordings paired with IMS scores	88	49	46	40	55.75 ± 21.8
	#Total days of recording	51	45	31	30	39.25 ± 10.4
	#Months over which recordings were done	5	5	3	5	4.5 ± 1
	IMS total score (mean ± std)	18.7 ± 3.8	−4.6 ± 6.4	26.4 ± 6.3	6.6 ± 7.6	

*F, Female; M, Male; UPDRS, Unified Parkinson’s Disease Rating Scale; off, 12 h off PD medication; on, on regular PD medication; BDI, Beck Depression Inventory; BAI, Beck Anxiety Inventory; MoCA, Montreal Cognitive Assessment; STN, Subthalamic Nucleus; GPi, Globus Pallidus interna; R, Right; L, Left; FPC, Frontopolar cortex; DLPFC, dorsolateral prefrontal cortex; OFC, orbitofrontal cortex; IMS, Immediate mood scaler, for assessing momentary mood state.*

*Contact coordinates are relative to the midcommissural point.

### Surgery

**FIGURE 1:**
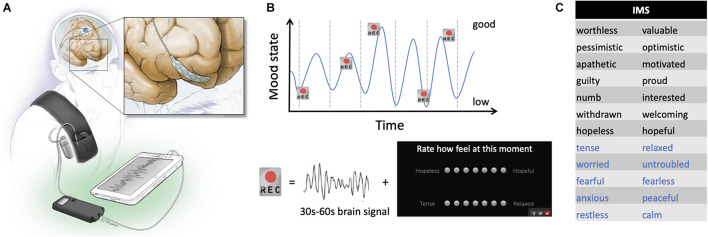
Device configuration and schematic of experimental recording paradigm. **(A)** Drawing of the electrodes and pulse generator *in situ*. The inset shows detail of the prefrontal recording lead. **(B)** Schematic representation of the protocol used. Brief brain recordings (“Rec”) were obtained over many days of mood fluctuations. Each recording consisted of 30–60 s of a bipolar time series from the prefrontal electrocorticography (ECoG) strip and was paired with patient self-report of symptoms using the Immediate Mood Scaler (IMS). **(C)** Items on the IMS for which study subjects provide scores on a visual analog scale. Black items relate to depression sub-scale; blue items relate to the anxiety subscale.

Subjects were implanted unilaterally or bilaterally with quadripolar DBS leads placed in either the subthalamic nucleus (STN; Medtronic Model 3389) or Globus Pallidus (GP); Medtronic Model 3387), according to clinical considerations (Table 1). Placement of the DBS lead was confirmed using microelectrode recordings in the awake state (Starr, 2002). In addition to the standard therapeutic DBS electrode(s) used to treat motor signs, patients were implanted with a flexible 4-contact electrocorticography (ECoG) lead (Medtronic 5387A) in the subdural space over the right PFC (Figure 1A). ECoG contacts were 4 mm in diameter and spaced 10 mm apart. The ECoG strips targeted the dorsolateral prefrontal cortex (DLPFC), the orbitofrontal cortex (OFC) or the frontopolar cortex (FPC), in order to evaluate a wide area of PFC in the course of this exploratory study. The cortical lead was placed through the original DBS burr hole in two patients. In two others, a second small burr hole was placed above the right orbit to access the OFC, which could not be accessed through the convexity burr hole due to the stiffness of the ECoG paddle. An intraoperative cone-beam CT merged to the preoperative MRI was used to confirm correct placement of the ECoG strip (Panov et al., 2017).

The cortical strip and ipsilateral DBS electrode were connected to lead extenders (model 37087, Medtronic), tunneled down the neck and attached to a Medtronic Activa PC+S pulse generator placed in a pocket over the pectoralis muscle under general anesthesia (Figure 1A). This investigational bidirectional device allows both delivery of therapeutic stimulation and chronic recording of field potentials (Stanslaski et al., 2018). For patients implanted with bilateral DBS electrodes, the left STN electrode was attached to a separate Activa SC pulse generator to deliver therapeutic stimulation.

### Electrode Locations and Tractography

To localize ECoG electrodes in individual patients, the preoperative T1 MRI was used to reconstruct cortical surface models in FreeSurfer (Dale et al., 1999; Fischl et al., 2002). A CT scan taken 2–3 months after surgery was used to determine the location of each cortical electrode. We projected ECoG contacts onto the cortical surface mesh with the imgpipe toolbox (Hamilton et al., 2017) using a surface vector projection method (Kubanek and Schalk, 2015). Once we identified cortical locations for each ECoG electrode on individualized cortical reconstructions, we projected all patients’ recording electrodes onto the Desikan-Killiany atlas brain (Desikan et al., 2006).

For tractography, we obtained High Angular Resolution Diffusion Imaging (HARDI) on a 3 Tesla MR scanner (General Electric, Inc.), using a spin-echo echo-planar imaging (SE EPI) pulse sequence (TE = 71 ms, TR = 7765 ms, flip angle alpha = 90°), FOV 28 cm × 28 cm, at least 70 axial slices, 2 mm^3^ isotropic voxels, *b*-value = 2000 s mm^–2^ in 55 non-collinear gradient directions and a signal to noise ratio >60. In PD1, *b*-value was 1,000 s mm^–2^ in 32 non-collinear gradient directions. A single non-diffusion-weighted b0 image was also obtained. The diffusion-weighted tractography was explored using a deterministic tractography software package (Brainlab Elements, Feldkirchen, Germany). Preoperative MRI, postoperative CT and HARDI scans were automatically merged and corrected for distortion. The ECoG contacts used for recordings were manually segmented on the CT scan. The regions of interest (ROIs) were then created by adding 2 mm to each contact and using these as seed regions for fiber tracking analyses, using an FA threshold of 0.17 and a minimum length of 8 cm.

### Experimental Design

Patients underwent chronic brain recording and monitoring of their symptoms during typical daily activities at home, over 3–5 months. Cortical recordings (30 or 60 s durations) were initially self-triggered, but patients subsequently found it easier to have the device automatically trigger neural recordings 2–3 times per day on a time schedule that was individualized to capture times of the day when low or high moods were typically experienced. Figure 1B shows a schematic representation of the paradigm used in this study. The recording montage was selected based on signal quality. Signals were sampled at 422 Hz, with a 0.5 Hz high pass filter, and a gain of 2,000. Signals were stored on the pulse generator and downloaded non-invasively by radiotelemetry during in-clinic research visits. Subjects were instructed to self-report their anxiety and depressive symptoms using the Immediate Mood Scaler (IMS, Posit Science) within a 30-min window of the time of the neural recordings. Assessments done without paired brain signal and outside that window were excluded from analysis, resulting in a variable number of recordings across patients (Table 1). The IMS is a validated tablet-based tool that assesses momentary mood symptoms (Nahum et al., 2017), correlates well with standardized self-report measures of depression (PHQ-9) and anxiety (GAD-7), and further captures symptom fluctuations in-the-moment. Subjects rated their current emotional state using 12 pairs of words thought to represent extremes of depressive (item 1–7, Figure 1C, black words) and anxiety (items 8–12, Figure 1C, blue words) related dimensions. The score range was −3 to 3 for each pair, with higher scores indicating more positive mood. Subjects were also instructed to assess the severity of their motor signs (rigidity, bradykinesia, tremor), their pain level, and the presence of suicidal thoughts using the same application (also on a score range of −3 to 3). We are not aware of a validated tool for self-assessment of motor signs at home. Changes in their basal ganglia DBS stimulation parameters, and changes in medications were occasionally required for clinical care and were tracked.

### Signal Processing and Statistics

Analyses were performed in Matlab. The first 2 s of each brain recording was discarded because of transient direct current offsets generated by the devices’ high-pass filter. For each brain recording, the power spectral density (PSD) was calculated using the Welch periodogram method (Matlab function pwelch) using a Hamming window, fast Fourier transform of 422 points and 50% overlap (frequency resolution of 1 Hz). The PSD was then log transformed and averaged over multiple frequency bands: 2–5 Hz delta, 5–8 Hz theta, 8–13 Hz alpha, 13–30 Hz beta, 13–20 Hz low beta, 20–30 Hz high beta, 30–45 Hz low gamma. For each patient, each frequency band was correlated with the total IMS score, or IMS subscores, using Spearman correlations because of discontinuous variables. A false-discovery rate (FDR) correction for multiple comparisons was used and a corrected *p*-value of 0.05 was considered statistically significant.

## Results

### Subjects

Four subjects with PD (3 females, 1 male) were studied. A fifth patient was implanted but did not did not provide regular symptom assessments and was thus excluded from further analyses. The mean age of study participants was 61 ± 7 years, mean disease duration was 11 ± 8 years and a mean UPDRS-III off medication score of 37 ± 13. Subjects had mild to moderate depression and anxiety (mean BDI score of 14 ± 5 and a mean BAI score of 16.5 ± 5). Patient demographics provided in Table 1.

### Recording Locations and Signals

**FIGURE 2:**
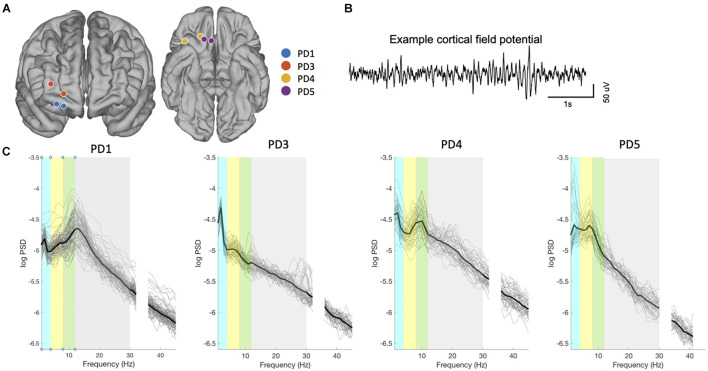
Contact localization and signal characteristics. **(A)** Localization of the ECoG electrodes used for bipolar recordings for all subjects, from fusion of postoperative CT scan to preoperative MRI scan, projected onto an atlas brain. Adjacent contacts were used for bipolar recording in 3 subjects (Contact spacing is 1 cm) and skipping one contact in PD4 (2 cm spacing). Spacing between contact appears variable here due to the projection of each electrode contact onto the Desikan-Killiany atlas brain, as described in the methods. **(B)** Example of raw signals from PFC (PD01). **(C)** Spectral properties of prefrontal neural signals (all recordings in all patients). Thin lines indicate power spectra from each recording, and thicker line denotes mean power spectra over all recordings from that subject. Colored bars indicate frequency bands: delta (blue), theta (yellow), alpha (green), beta (gray), gamma (white).

Chronic recordings through the prefrontal ECoG paddle were obtained while delivering therapeutic stimulation through the DBS lead (Figure 1A). In each subject, 40–89 recordings paired with symptom assessments were collected over a 3 to 5-month period beginning at least 10 days postoperatively (Table 1). The mean time between the brain recording and the mood assessment was 9 ± 10 min. Locations of the recording contacts used for each patient are represented on a template brain in Figure 2A, with coordinates in Table 1. An example ECoG time series is shown in Figure 2B. In all subjects, recordings were characterized by a spectral peak in theta, alpha, or beta frequencies as shown on Figure 2C, consistent with prefrontal ECoG time series in patients without PD (Helfrich and Knight, 2019).

### Self-Reported Mood States Are Correlated With Prefrontal Beta Band Activity

**FIGURE 3:**
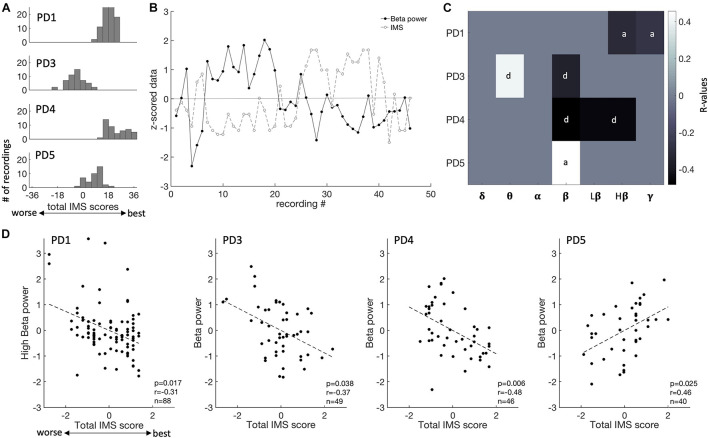
Mood state fluctuations and correlations of ECoG recordings with spectral features. **(A)** Distribution of IMS score for each patient (each row). IMS scores ranges from –36 to 36. **(B)** Fluctuation of IMS scores and beta power over the time course of this study (PD4). IMS scores and spectral power in each frequency band were normalized (z-scored) for ease of comparison within and across patients. **(C)** Summary of the correlation between all frequency bands and IMS total scores, showing *R*-values thresholded by *p*-values at a level of 0.05 [after false-discovery rate (FDR) correction]. Each patient is shown on a different row. The subscale that is the most predictive of mood is indicated (d, depression subscale; a, anxiety subscales). D, delta; T, theta; A, alpha; B, beta; LB, low beta; HB, high beta; G, gamma. **(D)** Beta spectral power correlations with total IMS score for each subject.

Patients’ symptoms were chronically assessed using the IMS, a tablet-based application that assesses momentary fluctuations of both anxiety and depressive symptoms with a total score ranging from −36 (worse) to 36 (best). These assessments, referred to here as “mood states,” fluctuated within ranges that were specific to each patient (Figure 3A and Table 1). We correlated spectral power in predefined frequency bands against total IMS scores and IMS subscores. An example of fluctuations in mood state as well as spectral power in the beta range is shown on Figure 3B (PD4). Across all four subjects, we found that beta power was a consistent predictor of mood states as shown in Figure 3C (*p* < 0.05, FDR corrected). In three subjects (PD1, 3 and 4), beta power was negatively correlated with total IMS scores (lower spectral power was associated with higher scores, corresponding to less anxiety and/or depression), while a positive correlation was found in PD5 (Figure 3D). One subject (PD3) also had a positive correlation between theta power and the IMS total score and another subject (PD1) had a negative correlation with gamma power (*p* < 0.05, FDR corrected, Figure 3C). In two subjects (PD3 and PD4) the correlation of beta activity with total IMS was driven mainly by depression subscores, while in the other two subjects (PD1 and PD5), IMS correlations were driven mainly by anxiety subscores.

### Recording Location May Explain Inverse Beta Correlation for PD5

**FIGURE 4:**
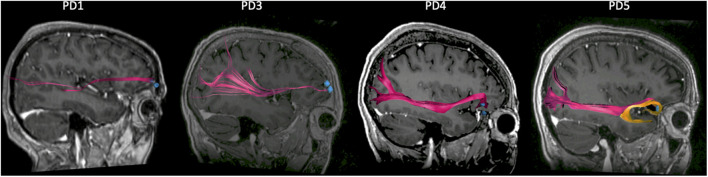
Connectivity of prefrontal recording locations from tractography. Sagittal view of the right hemisphere showing the large fiber tracts connecting the prefrontal cortex (PFC) to the occipital and parietal cortex (pink bundle), for each patient. PD5 differs from the other subjects in that an additional bundle (orange) connecting the PFC to the temporal lobe is present. The regions of interest (ROIs) corresponding to recording contact locations are indicated by the blue dots.

Since PD5 showed an opposite correlation between beta activity and IMS scores compared to other subjects, we sought to explain this based on contact location and connectivity to other brain regions. The recording montage for PD5 was the most mesial of all four subjects but did overlap with that of PD4 (Figure 2A). We thus used tractography to map the largest fiber tracts originating from the tissue immediately underneath the recording contacts in each subject. A seed object was made around each electrode contact in the bipolar recording montage. In all four subjects, the seed was the origin of fibers traveling in the inferior fronto-occipital fasciculus (IFOF), a pathway connecting widespread areas of PFC to the occipital and parietal cortex (Burks et al., 2018; pink bundle on Figure 4). The recording contacts in PD5, however, were uniquely associated with the uncinate fasciculus connecting mesial PFC to the temporal lobe (Hau et al., 2017; orange bundle on Figure 4), suggesting that recordings in this patient may have probed a different functional network compared to the other more laterally placed recording locations.

### Potential Confounding Variables

Given that symptoms were assessed at different times of the day, we studied the effect of time on symptoms assessment and found that the IMS scores were not correlated with the time at which they were done (*p* > 0.05, FDR corrected). In addition, since beta power is a marker of motor states in the basal ganglia nuclei, we also showed that cortical beta power did not covary with severity of motor signs (*p* > 0.05, FDR corrected). Thus, correlations between mood scores and cortical beta power were unlikely to have been confounded by these factors. While some changes to stimulation parameters and medications were made during the recording period to address clinical needs of the patient (Table 2), we did not observe consistent changes in self-ratings nor spectral power corresponding to the timing of those changes.

**TABLE 2:** Initial deep brain stimulator settings, medications, and their changes during the study.

Study subject	PD1	PD3	PD4	PD5
Initial DBS settings	C + 2-, 2.3 mV, 60 ms, 140 Hz	C + 2-, 2.5 V, 90 ms, 140 Hz	1 + 2-, 1.5 V 90 ms, 140 Hz	1–2 +, 3.2 V, 70 ms, 140 Hz
Changes in DBS settings during the study	Added additional contact: C + 1– 2-, and increased amplitude to 3.0 V at 88 days*	Increased amplitude to 3 V at 120 days*	Increased amplitude to 1.9 V at 30 days*	Changed stimulation contacts to 0 + 1 at 68 days*
Initial PD medications –levodopa equivalents	850	1,000	1,415	992
Change in PD levodopa equivalents	reduced to 650 at 38 days*	None	None	Increased to 1,192 at 68 days*
Initial psychiatric medication	Citalopram 20 mg	Lorazepam 1 mg	Duloxetine 20 mg Lexapro 20 mg Artane 2.5 mg	Duloxetine 90 mg QD
Change in psychiatric medication during the study	None	None	Lexapro decreased to 10 mg at 57 days*	Took mirtazapine 3.75 mg for 2 weeks starting at 113 days

*C, case of pulse generator (monopolar stimulation mode), V, Volts, ms, microseconds, Hz, Hertz; DBS, deep brain stimulation, PD, Parkinson’s disease.*

*Refers to the number of days since the start of brain recording.

## Discussion

We utilized chronic wireless invasive brain recording to elucidate the physiological basis for depression and anxiety in PD. We enrolled patients who met clinical criteria for basal ganglia DBS for motor signs of PD, who also had mild-to-moderate comorbid anxiety or depressive disorders. Over several months, prefrontal ECoG recordings were paired with patients’ self-reported mood scores. We show that variation in specific frequency bands, especially the beta band, explained part of the variance in mood scores. In recordings from DLPFC, FPC and lateral OFC, increased beta band activity correlated with worsening depression or anxiety symptom severity, while the opposite was true in the subject with the most mesially located contact pair in OFC. This is consistent with the anatomic localization of reciprocal reward and non-reward networks in the PFC (Rolls, 2016; Xia et al., 2017).

### Invasive Recording to Evaluate Non-motor Manifestations of Parkinson’s Disease

In movement disorders, specific *motor* signs have been related to alterations in oscillatory synchronization within and between structures of the basal ganglia-thalamocortical motor circuit. For example, beta band (13–30 Hz) oscillatory activity is exaggerated in the rigid-akinetic form of PD (Brittain and Brown, 2014), while gamma band (60–90 Hz) oscillations are elevated in levodopa-induced or stimulation-induced dyskinesia (Swann et al., 2016). The physiological signatures of non-motor symptoms, however, are much less understood. Invasive recording studies of the PFC (Chen et al., 2019) and STN (Kuhn et al., 2005; Buot et al., 2013; Huebl et al., 2014; Peron et al., 2017) have explored emotional functions of these brain regions, but only within task paradigms and during brief in-hospital sessions. EEG evaluation of PD patients with and without depressive symptoms showed differences in spectral power in alpha and low beta frequencies, but these were not localized to a particular brain region. This study is the first to evaluate cortical signatures of mood state in PD using invasive recording.

The method of chronic wireless recording from an implanted sensing-enabled interface was critical for tracking neural correlates of mood fluctuations over long periods during normal daily life. A disadvantage of this method, compared to perioperative ECoG with externalized leads (Panov et al., 2017; Sani et al., 2018), is lower channel count that precludes wide spatial coverage. To address this, we explored different prefrontal areas in each subject, and for statistical evaluation utilized a within-subjects repeated measures approach with over 40 samples per subject, rather than pooling data across subjects. Because the study took place over months, data were collected while clinically indicated therapeutic basal ganglia neurostimulation was also ongoing, which precluded acquiring simultaneous basal ganglia recordings (Swann et al., 2018). Recently introduced second generation sensing-enabled interfaces are engineered to allow depth recordings during stimulation (Gilron et al., 2021). We cannot exclude a possible effect of changes in medications or stimulation on cortical physiology, but changes were tracked and were infrequent. Only the right hemisphere was explored, based on safety consideration for an invasive investigational study, as well as evidence of greater mood-elevating effects of acute prefrontal stimulation on the right side, in non-parkinsonian disorders (Rao et al., 2018).

### Opposite Beta Correlations May Reflect Reciprocal Roles of Two Reward-Related Networks

Our findings suggest that beta band activity may index the severity of non-motor symptoms in networks linked to the PFC. There are thought to be two reciprocally related cortico-striatal reward processing networks involving the PFC (Rolls, 2016; Xia et al., 2017). A “non-reward” (missed reward) network, whose cortical localization maps to lateral OFC, DLPFC, and dorsal cingulate cortex, is activated when an action does not lead to positive reward. A positive reward network, involving mesial PFC and pregenual cingulate cortex, is active when action *does* lead to a reward. The mesial reward network also has major connections to the temporal lobe (Loonen and Ivanova, 2016a,b). Consistent with this framework, and with the possibility that depressed mood may reflect dysfunctional reward networks, non-invasive studies indicate that functional connectivity (resting state MRI fluctuations) in these reciprocal cortical areas have opposite correlations with severity of depression (in non-parkinsonian mood disorders) (Cheng et al., 2016). Our results from this initial invasive recording study in PD support this dichotomous circuit model, showing elevated beta in states of depressed mood or anxiety in the lateral OFC and DLPFC, and the inverse in mesial OFC. Of note, while the two subjects with OFC recording leads had overlapping recording montages, DTI-based tractography showed differing connectivity of these montages. Only those from PD5 (the only subject to have increased beta activity during epochs of *better* mood), showed temporal lobe connectivity consistent with involvement in the mesial positive reward network.

### Beta Activity and the Maintenance of Attractor States

In the normal function of *motor* networks, beta band oscillatory activity is thought to favor “maintenance of the *status quo*,” that is, elevated while holding specific postures, and desynchronizing for fluid movements to occur (Engel and Fries, 2010). By analogy, in PFC, we hypothesize that beta oscillations could function to maintain specific affective states, depending on the region of the PFC involved. This hypothesis dovetails with the recently proposed “non-reward attractor” theory of depression, postulating that depression can be triggered by abnormally prolonged activation of the lateral prefrontal network sensitive to missed or absent reward (Rolls, 2016). Beta band activity thus might serve to maintain “attractor states”: increased beta in lateral OFC and DLPFC could contribute to maintenance of a “depressive” attractor state, while increased beta in mesial OFC would promote a “rewarding” or mood-elevating attractor state. Given the strong influence of dopamine depletion and dopaminergic medications on mood and anxiety (Seppi et al., 2019), it is possible that our findings are specific to patients with PD and would not generalize to mood disorders in non-parkinsonian states. However, in patients with major depressive disorder undergoing placement of DBS leads in the subgenual cingulate (part of the mesial reward network), severity of depression across subjects inversely correlated with the amplitude of beta band oscillations (Clark et al., 2016), as in subject PD5 in this study, suggesting a role for beta activity in maintenance of reward networks across multiple diagnoses.

### Implications for Treatment of Non-motor Signs of Parkinson’s Disease

Depression, anxiety, and behavioral changes in PD may be difficult to treat and may become a primary source of disability in mid-stage PD after motor signs are adequately treated medically or by surgical intervention (Schupbach et al., 2006). Our work provides a framework for therapeutic neuromodulation of beta activity in non-motor networks in PD. Non-invasive methods of neuromodulation of DLPFC show promise for mood disorders in PD (Rektorova and Anderkova, 2017). Subcortical intervention, where fibers converge on smaller targets, may be more efficient for modulating mood networks. Theta burst stimulation of ventral STN, for example, has been used to modulate theta band activity in DLPFC (Bentley et al., 2020). STN stimulation paradigms that reduce lateral prefrontal beta activity merit investigation and could be facilitated by the enhanced spatial resolution offered by recently introduced “directional” DBS leads (Aman et al., 2020).

## Data Availability Statement

The raw data supporting the conclusions of this article will be made available by the authors, without undue reservation.

## Ethics Statement

The studies involving human participants were reviewed and approved by the Institutional Review Board of the University of California, San Francisco. The patients/participants provided their written informed consent to participate in this study.

## Author Contributions

CH, EC, HD, and PS: conception and design of the study. CH, WC, CR, AS, AM, MY, SW, RG, SL, IB, MS, MK, JO, and PS: acquisition of data. CH, WC, RG, and SL: analysis of data. CH, WC, and PS: drafting a significant portion of the manuscript or figures. All authors contributed to the article and approved the submitted version.

## Conflict of Interest

PS receives research support from Medtronic Inc., (investigational implantable devices utilized here were provided at no charge). PS and JO receive funding for fellowship training from Medtronic Inc. The remaining authors declare that the research was conducted in the absence of any commercial or financial relationships that could be construed as a potential conflict of interest.

## Publisher’s Note

All claims expressed in this article are solely those of the authors and do not necessarily represent those of their affiliated organizations, or those of the publisher, the editors and the reviewers. Any product that may be evaluated in this article, or claim that may be made by its manufacturer, is not guaranteed or endorsed by the publisher.

## Footnotes

1
clinicaltrials.gov/ct2/show/NCT03131817
